# Vitamin D levels and clinical outcomes of SARS-CoV-2 Omicron subvariant BA.2 in children: A longitudinal cohort study

**DOI:** 10.3389/fnut.2022.960859

**Published:** 2022-07-25

**Authors:** Denggao Peng, Hua Huang, Zhichao Liu, Yanzhang Gao, Yingxia Liu

**Affiliations:** ^1^Department of Emergency Medicine, Shenzhen Third People's Hospital, Second Hospital Affiliated to Southern University of Science and Technology, Shenzhen, China; ^2^Graduate Collaborative Training Base of Shenzhen Third People's Hospital, Hengyang Medical School, University of South China, Hengyang, China; ^3^Department of Radiology, Shenzhen Third People's Hospital, Second Hospital Affiliated to Southern University of Science and Technology, Shenzhen, China

**Keywords:** children, Omicron, subvariant, SARS-CoV-2, vitamin D, BA.2

## Abstract

**Objective:**

To investigate the picture between vitamin D levels and clinical outcomes of SARS-CoV-2 Omicron subvariant BA.2 in children.

**Methods:**

A retrospective, longitudinal cohort study was performed. All included hospitalized cases were divided into the sufficient (sVD) and insufficient vitamin D (iVD) groups according to whether their serum 25-hydroxyvitamin D [25(OH)D] concentration was ≥30 ng/mL. Dynamic changes in clinical parameters were observed for seven time periods within 28 days after admission.

**Results:**

Serum 25(OH)D concentrations were significantly negatively correlated with age in the included cases (*r* = −0.6; *P* < 0.001). Compared with the iVD group (*n* = 80), the sVD group (*n* = 36) had higher interleukin-6 (18.4 vs. 12.9; *P* = 0.003) within the first day; higher procalcitonin within the first (0.15 vs. 0.1; *P* = 0.03), 2–3 (0.14 vs. 0.07; *P* = 0.03), 4–5 (0.21 vs. 0.07; *P* = 0.02) days; more lymphocytes within the first (1.6 vs. 1.2; *P* = 0.02), 2–3 (3.7 vs. 2; *P* = 0.001), 4–5 (3.9 vs. 2.1; *P* = 0.01) and 6–7 (4.9 vs. 2.7; *P* = 0.02) days; notably, higher cycle threshold for N gene (30.6 vs 19.8; *P* = 0.03) or ORF1ab gene (31.4 vs 20.1; *P* = 0.03) within 2 to 3 days. Pneumonia lesions were found in eleven and six cases in the iVD and sVD groups, respectively, without significant difference on computed tomography at admission. Six out of eleven and five out of six had a repeat computed tomography after 1–2 weeks. Lesion improvement was more significant in the sVD group (*P* = 0.04).

**Conclusions:**

Children with vitamin D insufficiency might have poorer clinical outcomes in Omicron subvariant BA.2 infection, especially in older pediatric patients. Further studies are needed to assess effectiveness of supplements in reducing the same.

## Introduction

Since the outbreak of coronavirus disease 2019 (COVID-19) caused by severe acute respiratory syndrome coronavirus 2 (SARS-CoV-2) in late 2019, five variants including Alpha, Beta, Gamma, Delta and Omicron have been designated by the World Health Organization as variants of concern (1). To date, the Omicron variant has become the overwhelmingly dominant strain worldwide, with virological, epidemiological and clinical characteristics significantly different from the ancestral virus (2, 3). *In vitro* antibody neutralizing assays showed that the Omicron variant could partially evade serum neutralizing activity in SARS-CoV-2 vaccinees or convalescents after infection with the original strain or other variants (4–6). Furthermore, it has been reported that the Omicron variant replicates actively in the upper airways, giving it the ability to transmit efficiently and have a short doubling time, but is less invasive in the lower airways (2). The immune system and respiratory barrier function of children are immature. Our previous study also found immunosuppression and delayed viral clearance in children infected with wild-type SARS-CoV-2 compared with adults (7). Even more unfortunate is that children have very low vaccinations and poor adherence to epidemic prevention. Therefore, the prevention and control of pediatric Omicron infection is difficult and worrying. The development of Omicron-specific vaccines along with boosting vaccinations or immunomodulation could be very promising for improving clinical outcomes of Omicron infection in children.

As a steroid pro-hormone that maintains calcium and phosphorus homeostasis, vitamin D has immunomodulatory properties by stimulating the expression of innate and adaptive immune function receptors in airway epithelial cells to comprehensively prevent acute respiratory infections (8–10). In addition, vitamin D can downregulate pro-inflammatory cytokines and balance the inflammatory response, thereby avoiding the cytokine storm-induced host tissue damage during acute viral infection (11, 12). Despite some controversy and inconsistency (13, 14), multiple studies have suggested that vitamin D may reduce COVID-19 mortality and severity in hospitalized patients, especially in adults with vitamin D deficiency (15, 16). Notably, a recent study revealed an association between vitamin D deficiency and clinical severity and inflammatory markers in pediatric COVID-19 cases, and recommended preventive vitamin D supplementation (17).

The Omicron variant consists of several phylogenetically related sublineages, such as BA.1, BA.2, BA.3, but with very different spike protein sequences (2, 18). BA.1 is the predominant sublineage prevalent globally, but the proportion of BA.2 has skyrocketed since January 2022 (2, 19). Vaccine development and clinical studies targeting Omicron are primarily based on BA.1, while little is known about the clinical characteristics and outcomes of BA.2 and the specific effects of vitamin D against BA.2 infection. In particular, children are a special population at high risk for vitamin D insufficiency (20), and data on the picture between vitamin D levels and clinical outcomes of SARS-CoV-2 Omicron subvariant BA.2 have not been reported. We conducted a retrospective, longitudinal cohort study to provide evidence for epidemic prevention and control in children.

## Materials and methods

### Clinical definition and classification

Children were defined as being <18 years old. Quantitative reverse transcriptase-polymerase chain reaction (qRT-PCR) was used to detect SARS-CoV-2 positive and viral load in nasopharyngeal swab samples. A cycle threshold of <40 for N gene or ORF1ab gene was defined as positive. Whole-genome sequencing and bioinformatics analysis were used to confirm SARS-CoV-2 variant types and sublineages. SARS-CoV-2 IgM, IgG were measured by using a chemiluminescence method. All children diagnosed with COVID-19 were admitted to Shenzhen Third People's Hospital for isolation and treatment, and relevant examinations were completed as routine procedures. Fever was recognized when body temperature is higher than or equal to 37.3°C. Respiratory symptoms included nasal congestion, runny nose, sneezing, sore throat, cough, expectoration, chest pain, and dyspnea. Digestive symptoms included nausea, vomiting, abdominal pain, and diarrhea.

Serum 25-hydroxyvitamin D [25(OH)D] concentrations were measured by an immunoinhibition assay kit. We classified serum 25(OH)D levels into two groups with reference to cut-off points commonly used in Global Consensus Recommendations: (1) the insufficient vitamin D (iVD) group was defined as serum 25(OH)D concentration <30 ng/mL; (2) the sufficient vitamin D (sVD) group was defined as serum 25(OH)D concentration ≥30 ng/mL (21).

The COVID-19 pneumonia lesions on chest computed tomography (CT) included ground glass opacities (GGOs), consolidation and nodular opacities. GGOs were defined as hazy areas of increased opacity or attenuation without concealing the underlying vessels. Consolidation was defined as homogeneous opacification of the parenchyma with or without surrounding halo sign. Nodular opacities were defined as focal round opacities (22).

### Imaging evaluation

Non-contrast thin-section chest CT was performed on each pediatric patient using Shanghai uCT760 64-row spiral CT machine (reconstruction slice thickness 0.625 mm). All image data were observed within the lung window with a window settings (width 1600 HU; level−550 HU).

The COVID-19 pneumonia lesions were automatically identified and quantified using artificial intelligence (AI) software (InferRead CT Pneumonia, V1.1.3.0, Tuixiang, Beijing, China), followed by calculation of total lesion volume (sum volume of GGO, consolidation and nodular opacities) and simultaneous total lung volume. Meanwhile, two experienced pediatric radiologists independently reviewed all chest CT images and manually calculated the total lesion volume. They were blinded to the clinical information and conditions, except for the knowledge that these were cases of SARS-CoV-2 infection. The average of three measurements (1 AI, 2 manual) was determined as the final total lesion volume. The ratio of total lesion volume to total lung volume was calculated for comparison under different conditions.

### Data collection and review

**Figure 1:**
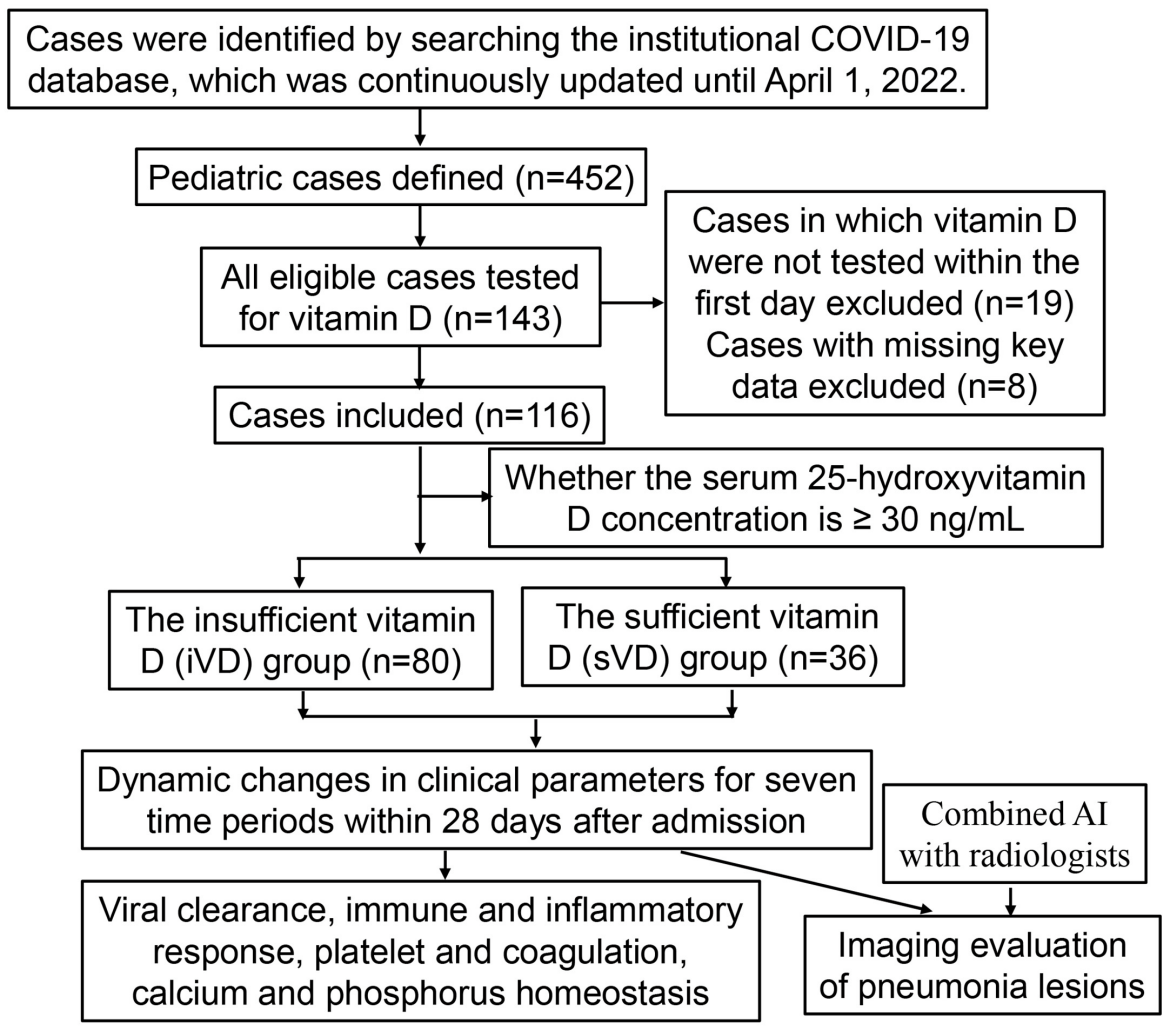
Flow chart of the pediatric cases selection and analysis process.

The institutional COVID-19 database of Shenzhen Third People's Hospital was used, which was continuously updated until April 1, 2022. We retrieved electronic medical records and conducted a retrospective study. The clinical and laboratory data of all COVID-19 children during hospitalization were collected and reviewed. Based on the results of serum 25(OH)D concentration, whole genome sequencing and bioinformatics analysis, all included pediatric cases infected with Omicron subvariant BA.2 were divided into the iVD and sVD group. Given that the median [interquartile range (IQR)] length of hospital stay was 22 (18–24) days, we selected seven time periods from T_1_ to T_7_ within 28 days after admission to observe dynamic changes in clinical parameters. T_1_ to T_7_ represent the following: T_1_ ≤ 1 day; 1 < T_2_ ≤ 3 days; 3 < T_3_ ≤ 5 days; 5 < T_4_ ≤ 7 days; 7 < T_5_ ≤ 14 days; 14 < T_6_ ≤ 21 days; 21 < T_7_ ≤ 28 days. The ratios of total lesion volume to total lung volume on CT were compared between the iVD and sVD groups at admission and 1–2 weeks after admission, respectively. The case selection process was presented in Figure 1.

Inclusion criteria:

(1) As of April 1, 2022, all discharged pediatric cases who were infected with Omicron subvariant BA.2 and tested for serum 25(OH)D concentration during hospitalization.

Exclusion criteria:

(1) Cases in which serum 25(OH)D concentrations were not tested within the first day after admission.
(2) Cases with missing key data.

### Statistical analysis

All analyses were conducted by using of IBM Statistical Product and Service Solutions Version 26 (SPSS Inc, Chicago, IL) and GraphPad Prism 8 software. Continuous variables were summarized as the median with IQR or mean with standard deviation (SD), median (IQR) or (mean ± SD), depending on whether their distributions were normal or not. Comparisons of categorical variables were performed using the Pearson Chi-square test or Fisher exact test. Two-tailed Pearson or Spearman correlation analysis was performed to evaluate the correlation between two continuous variables. The parametric tests (independent sample Student *t*-test) or non-parametric tests (Mann-Whitney U test) were used to analyze variables. *P* < 0.05 was considered as statistically significant in all tests if applied.

## Results

### Demographic and clinical characteristics

**Figure 2:**
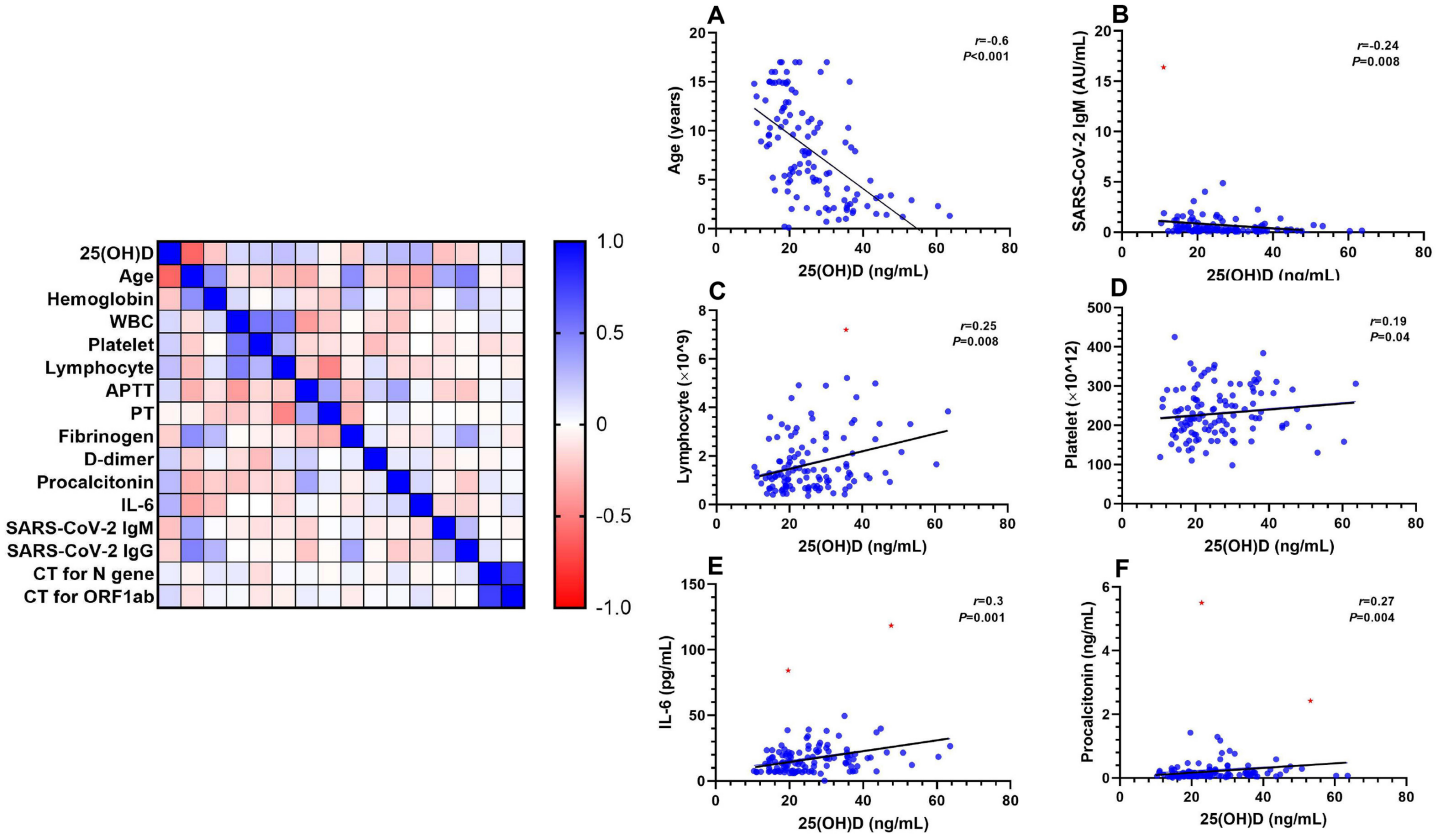
Spearman correlation analysis of serum 25(OH)D concentrations with other clinical parameters, *r* represents the correlation coefficient. The red dots in B, C, E, F represent outliers. APTT, activated partial thromboplastin time; CT, cycle threshold; IL-6, interleukin-6; PT, prothrombin time; WBC, white blood cell. **(A)** age; **(B)** SARS-CoV-2 IgM; **(C)** lymphocytes; **(D)** platelets; **(E)** IL-6; **(F)** procalcitonin. Equation: **(A)** Y = −0.276*X + 15.1; **(B)** Y = −0.03*X + 1.51 (Outlier included); Y = −0.012*X + 0.88 (Outlier excluded); **(C)** Y = 0.035*X + 0.81 (Outlier included); Y = 0.031*X + 0.86 (Outlier excluded); **(D)** Y = 0.76*X + 212; **(E)** Y = 0.38*X + 7.85 (Outliers included); Y = 0.25*X + 9.8 (Outliers excluded); **(F)** Y = 0.006*X + 0.11 (Outliers included); Y = 0.003*X + 0.13 (Outliers excluded).

As of April 1, 2022, a total of 116 discharged pediatric patients with Omicron subvariant BA.2 infection were included in this study, of whom 65 (56%) were male. Median (IQR) for age and serum 25(OH)D concentration were 7.8 (3.3–12) years and 23.9 (18.6–31.4) ng/mL, respectively. Serum 25(OH)D concentrations were significantly negatively correlated with age (*r* = −0.6; *P* < 0.001) (Figure 2A). Detailed demographic characteristics were presented in Table 1.

**Table 1:** Demographic characteristics of pediatric cases infected with Omicron subvariant BA.2.

**Study population**	***n*** = **116**
Age (years): Median (IQR)	7.8 (3.3–12.0)
≤3: *n* (%)	26 (22.4%)
>3, ≤6: *n* (%)	25 (21.6%)
>6, ≤12: *n* (%)	37 (31.9%)
>12, ≤18: *n* (%)	28 (24.1%)
Male gender: *n* (%)	65 (56.0%)
Serum 25(OH)D concentration (ng/mL): Median (IQR)	23.9 (18.6–31.4)

*25(OH)D, 25 hydroxyvitamin D; IQR, interquartile range*.

All included pediatric cases were divided into the iVD group (*n* = 80) and the sVD group (*n* = 36). The sVD group was younger than the iVD group [2.9 (1.9–4.5) vs. 9.7 (5.9–13.7); *P* < 0.001], while differences in percentage of male gender (53 vs. 58%), vaccination ratio (14 vs. 21%) and imaging findings of pneumonia (17 vs. 14%) were not statistically significant. There was no patient who was severely ill. Three (3.8%) cases in the iVD group and four (11%) cases in the sVD group were asymptomatic; 38 (48%) and 22 (61%) presented with fever; 38 (48%) and 11 (31%) presented with respiratory symptoms; 10 (13%) and four (11%) presented with digestive symptoms. There was no statistical difference between the two groups. Detailed univariate comparison of baseline clinical characteristics were presented in Table 2.

**Table 2:** Univariate comparison of clinical characteristics between the iVD and sVD groups.

	**iVD group (*****n** =* **80)**	**sVD group (*****n** =* **36)**	***P***
Age (years): Median (IQR)	9.7 (5.9–13.7)	2.9 (1.9–4.5)	<0.001
Male gender: *n* (%)	46 (57.5%)	19 (52.8%)	0.689
Vaccination: *n* (%)	17 (21.3%)	5 (13.9%)	0.447
Clinical symptoms: *n* (%)			
Asymptomatic	3 (3.8%)	4 (11.1%)	0.201
Fever	38 (47.5%)	22 (61.1%)	0.229
Respiratory symptoms	38 (47.5%)	11 (30.6%)	0.106
Digestive symptoms	10 (12.5%)	4 (11.1%)	1.000
Imaging findings of pneumonia: *n* (%)	11 (13.8%)	6 (16.7%)	0.778
Positive SARS-CoV-2 IgM: *n* (%)	1 (1.3%)	2 (5.6%)	0.227
Positive SARS-CoV-2 IgG: *n* (%)	56 (70.0%)	18 (50.0%)	0.059

*25(OH)D, 25 hydroxyvitamin D; iVD, insufficient vitamin D; IQR, interquartile range; sVD, sufficient vitamin D; SARS-CoV-2, severe acute respiratory syndrome coronavirus 2*.

### Viral clearance

**Figure 3:**
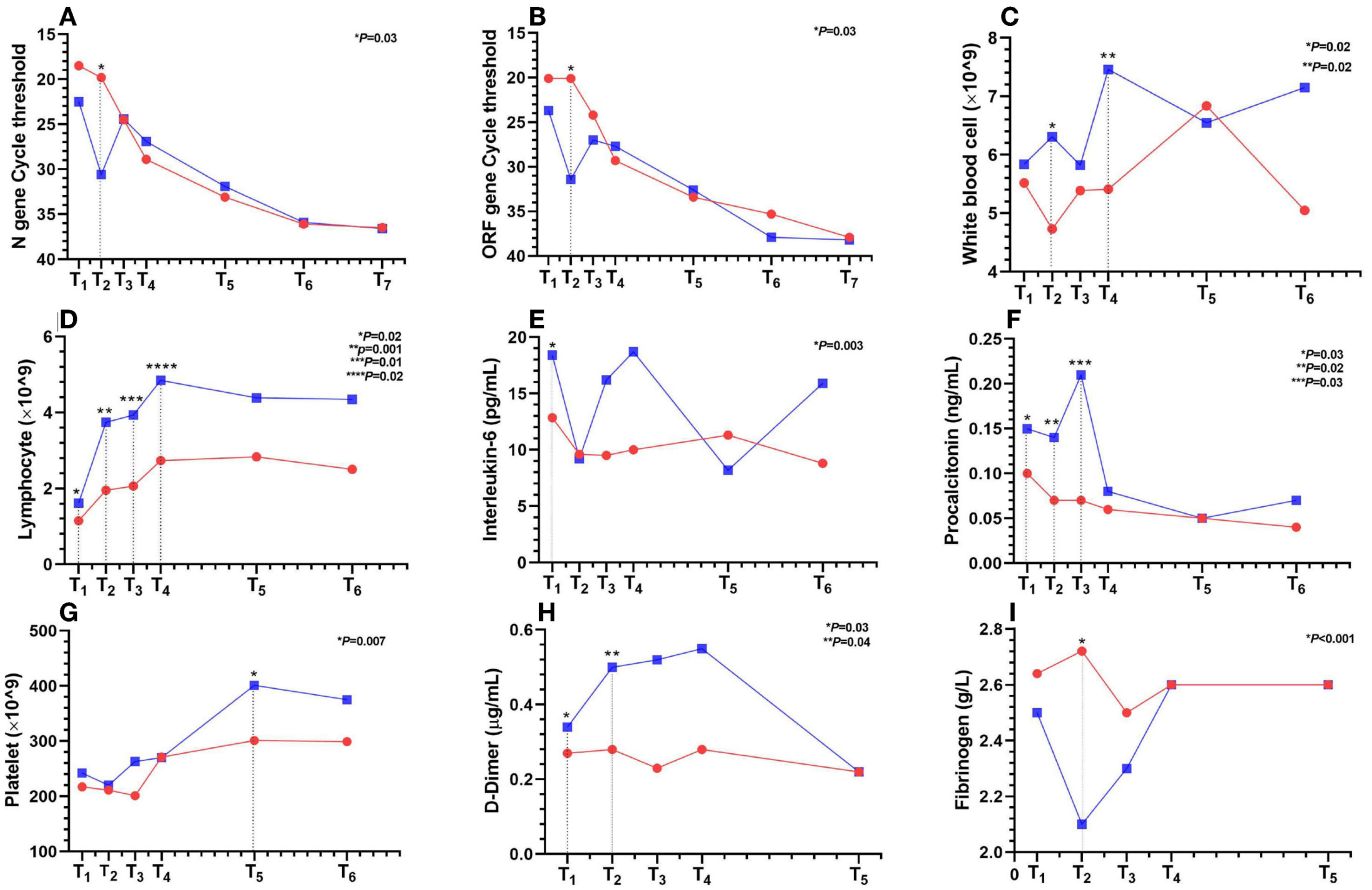
Dynamic changes in laboratory results for seven time periods from T_1_ to T_7_ within 28 hospitalization days after admission. T_1_ to T_7_ represent the following: T_1_ ≤ 1 day; 1 < T_2_ ≤ 3; 3 < T_3_ ≤ 5; 5 < T_4_ ≤ 7; 7 < T_5_ ≤ 14; 14 < T_6_ ≤ 21; 21 < T_7_ ≤ 28 days. The red line represents the iVD group, *n* = 8–80 per condition. The blue line represents the sVD group, *n* = 4–36 per condition. Points on the line chart represent mean or median. **(A)** N gene cycle threshold; **(B)** ORF1ab gene cycle threshold; **(C)** white blood cells; **(D)** lymphocytes; **(E)** interleukin-6; **(F)** procalcitonin; **(G)** platelets; **(H)** D-dimer; **(I)** fibrinogen.

Serum 25(OH)D concentrations were not significantly correlated with cycle thresholds for N gene (Supplementary Figure S1A) or ORF1ab (Supplementary Figure S1B) gene within the first day after admission. While the sVD group had higher cycle threshold for N gene (30.6 vs 19.8; *P* = 0.03) (Figure 3A) or ORF1ab gene (31.4 vs 20.1; *P* = 0.03) (Figure 3B) within 2 to 3 days.

### Immune response

One (1.3%) case in the iVD group and two (11%) cases in the sVD group were positive for SARS-CoV-2 IgM, 56 (70%) and 18 (50%) positive for SARS-CoV-2 IgG at admission. There was no statistical difference between the two groups (Table 2). Serum 25(OH)D concentrations were negatively correlated with serum SARS-CoV-2 IgM titers (*r* = −0.24; *P* = 0.008) (Figure 2B), but not IgG titers (Supplementary Figure S1C). There was no significant difference in SARS-CoV-2 IgM and IgG titers. Five (14%) cases in the iVD group and 17 (21%) cases in the sVD group had received either one or two doses of SARS-CoV-2 vaccine. There was also no significant difference in IgG titers among vaccinated children (Supplementary Figures S2A–C).

### Inflammatory response

Serum 25(OH)D concentrations were positively correlated with lymphocytes (*r* = 0.25; *P* = 0.007) (Figure 2C) or interleukin-6 (IL-6) (*r* = 0.3; *P* = 0.001) (Figure 2E) or procalcitonin (*r* = 0.27; *P* = 0.004) (Figure 2F), but not correlated with white blood cells (WBCs) (Supplementary Figure S1D). Compared with the iVD group, the sVD group had higher IL-6 (18.4 vs. 12.9; *P* = 0.003) within the first day (Figure 3E); higher procalcitonin within the first (0.15 vs. 0.1; *P* = 0.03), 2–3 (0.14 vs. 0.07; *P* = 0.03), 4–5 (0.21 vs. 0.07; *P* = 0.02) days (Figure 3F); more lymphocytes within the first (1.6 vs. 1.2; *P* = 0.02), 2–3 (3.7 vs. 2; *P* = 0.001), 4–5 (3.9 vs. 2.1; *P* = 0.01) and 6–7 (4.9 vs. 2.7; *P* = 0.02) days (Figure 3D); and higher WBCs within 2–3 (6.3 vs. 4.73; *P* = 0.02), 6–7 (7.46 vs. 5.41; *P* = 0.02) days (Figure 3C).

### Platelet and coagulation

Serum 25(OH)D concentrations were positively correlated with platelets (*r* = 0.19; *P* = 0.04) (Figure 2D), but not correlated with activated partial thromboplastin time (APTT) (Supplementary Figure S1F) or prothrombin time (PT) (Supplementary Figure S1I) or fibrinogen (Supplementary Figure S1H) or D-dimer (Supplementary Figure S1G). Compared with the iVD group, the sVD group had higher D-dimer (0.5 vs. 0.28; *P* = 0.046), and lower fibrinogen (1.99 vs. 2.72; *P* < 0.001) within 2–3 days (Figures 3H,I); longer APTT (44.2 vs. 40; *P* = 0.01) within 3–5 days (Supplementary Figure S2F); higher platelet count (400 vs. 300; *P* = 0.007) within 8–14 days (Figure 3G). There was no significant difference in PT (Supplementary Figure S2G).

### Calcium and phosphorus homeostasis

Serum calcium and phosphorus in the sVD group tended to be higher than those in the iVD group within 28 days after admission, and serum calcium in the sVD group was significantly higher within 8–14 (2.52 vs. 2.38; *P* = 0.03), and 15–21 days (2.46 vs. 2.41; *P* = 0.03) (Supplementary Figure S2H). There was no significant difference in serum phosphorus (Supplementary Figure S2I). All detailed data were presented in Supplementary Tables S1, S2.

### Imaging evaluation of pneumonia lesions

**Figure 4:**
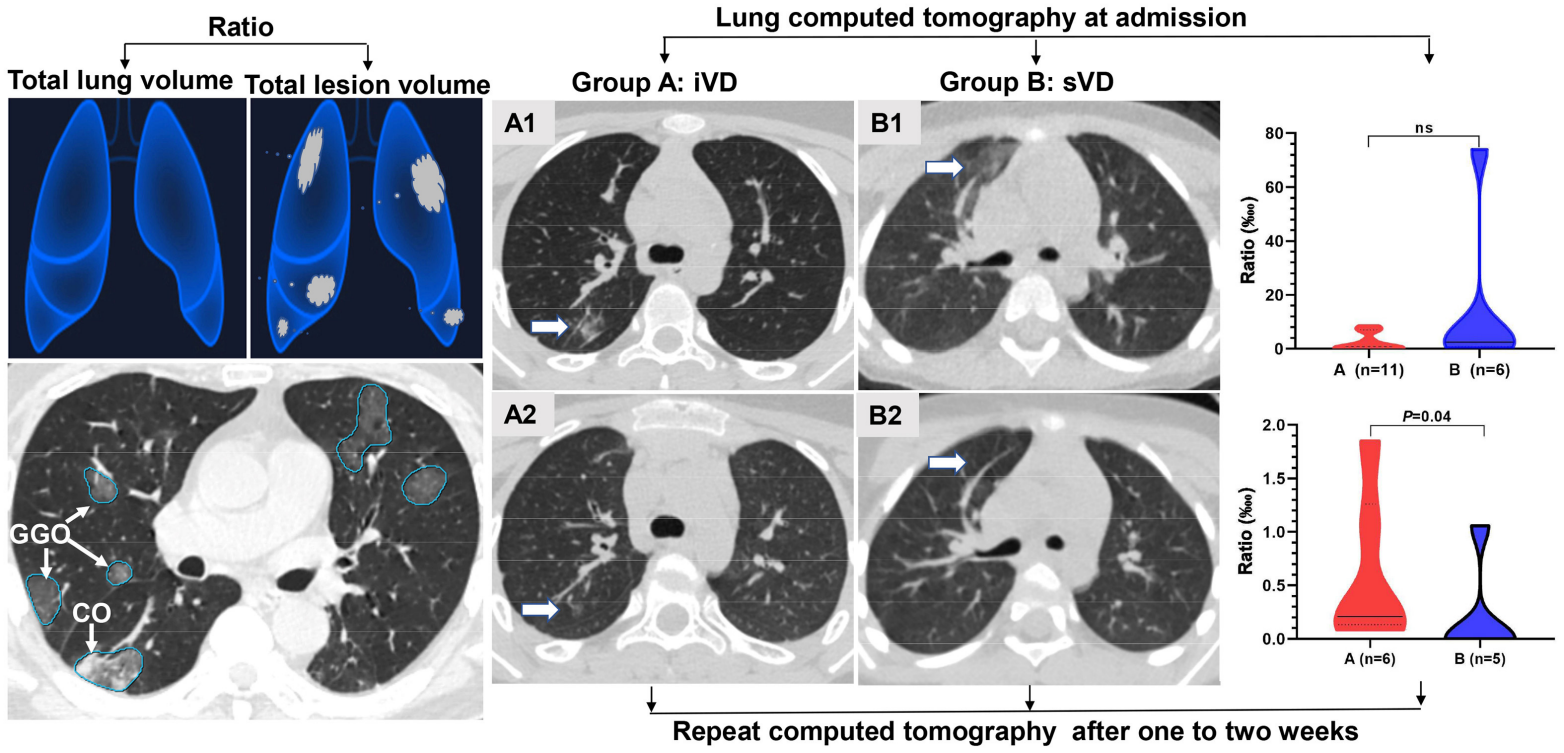
The COVID-19 pneumonia lesions included ground-glass opacity (GGO), consolidation (CO), and nodular opacities were identified and measured by AI and radiologists. The differences in the ratio of total lesion volume to total lung volume in computed tomography at admission (not significant) and 1–2 weeks later (*P* = 0.04) were compared between the iVD (A) and sVD (B) groups.

Pneumonia lesions mainly including GGOs and consolidation were found in eleven (14%) and six (17%) cases in the iVD and sVD groups, respectively (Table 2). There was no significant difference in the ratios of total lesion volume to total lung volume on CT at admission. Six out of eleven and five out of six had a repeat CT after 1–2 weeks. Lesion improvement characterized by the reduced ratio of total lesion volume to total lung volume was more significant in the sVD group (*P* = 0.04) (Figure 4).

## Discussion

To our knowledge, this is the first longitudinal cohort study on the picture between vitamin D levels and clinical outcomes of SARS-CoV-2 Omicron subvariant BA.2 in children so far. Our observational study highlighted the findings that vitamin D sufficiency, with a serum 25(OH)D concentration of at least 30 ng/mL, may accelerate viral clearance early in the course of COVID-19, subsequently improve pneumonia lesions, and ultimately improve clinical outcomes of Omicron subvariant BA.2 in children. The protective effect of vitamin D sufficiency may be achieved by modulating immunity, reducing lymphocyte depletion, and balancing inflammatory responses. We innovatively combined AI with radiologists to identify and measure the dynamic changes of pneumonia lesions, which solved the problem that Omicron-infected pneumonia is difficult to quantify and is not conducive to comparison under different conditions.

The underlying viral clearance mechanism in children is still not completely understood. CD4+ T lymphocytes are the center of the immune system, helping in the production of antibodies. CD8+ T lymphocytes mediate cytotoxic immune responses and play a vital role in viral clearance and immune-related inflammation (7, 23). In our investigation, serum 25(OH)D concentrations were positively correlated with lymphocytes or IL-6 or procalcitonin, in part consistent with related reports (15, 17). And within 1 week after admission, lymphocytes in the two groups gradually increased synchronously; IL-6 in the sVD group gradually decreased, and that in the iVD group gradually increased; procalcitonin in the sVD group initially increased and then decreased, and that in the iVD group gradually decreased. But at the same time period, the three parameters in the sVD group were almost higher than those in the iVD group. Interestingly, the cycle threshold for N gene or ORF1ab gene in the sVD group, which were inversely related to viral load, were consistently higher than those in the iVD group, during the first 5 days after admission, with significant differences within 2–3 days. These results suggest that vitamin D sufficiency may accelerate viral clearance by modulating cellular immunity, reducing lymphocyte depletion, and balancing inflammation. Although lymphocyte subclassification was not performed, we speculate that the depletion of mainly CD8+ T lymphocytes was reduced. Immunomodulation targeting CD8+ T lymphocytes may be a promising treatment for Omicron infection.

Moreover, our study found that vitamin D insufficiency was common in the included pediatric cases, and serum vitamin D concentrations were significantly negatively correlated with age. The cause of vitamin D insufficiency in older children may be related to insufficient sunshine exposure and neglect of additional vitamin D supplements. Vitamin D insufficiency may negate the benefits of a child's immune system maturing with age. Therefore, additional vitamin D supplementation in pediatric patients infected with Omicron may be very necessary, especially in older children.

A study have shown that vitamin D enhances CD4+ T lymphocytes in adaptive immunity and promotes the production of virus-specific antibodies by activating T lymphocyte-dependent B lymphocytes (23). However, our investigations observed inconsistent changes that serum vitamin D concentrations were negatively correlated with SARS-CoV-2 IgM titers. Furthermore, vitamin D sufficiency did not affect SARS-CoV-2 IgG titers in vaccinated or all included children. Notably, the viral antigens currently used in the clinical serological detection of SARS-CoV-2 antibodies are basically developed using the original strain through multiple technical routes (18). However, the antigenic site in the spike protein receptor binding domain (RBD) of Omicron subvariant BA.2 has undergone multiple mutations, and viral RBD-specific antibodies have very different conformations (2, 6, 24). As a result, the ability of these original strain antigens to capture Omicron variant-specific antibodies has been greatly reduced (4). In addition, the serological detection parameters of SARS-CoV-2 antibodies, such as reference range, threshold, sensitivity, and regularity of antibody production, are all based on big data from adult clinical trials. Children have very low vaccination and corresponding laboratory data are relatively lacking. There are questions about the practicality and suitability of using adult testing standards for children. Overall, whether vitamin D sufficiency promotes the production of Omicron subvariant BA.2-specific antibodies may require further validation by developing Omicron-specific vaccines and antigens, and designing large-scale clinical trials in children. The good news is that an Omicron-specific vaccine in China has entered clinical trials and is expected to be widely available for vaccination by the end of this year.

The coagulation cascade is activated during viral infections. This response may be part of the host's defense system to limit spread of a pathogen (25). Bayramoglu et al. found that vitamin D levels were inversely correlated with fibrinogen levels in pediatric cases, and the moderate-severe group had higher D-dimer (17). We have also observed similar changes. Compared with the iVD group, the sVD group had relatively higher D-dimer and lower fibrinogen, which were statistically different within 2–3 days after admission. This suggests that activation of coagulation may be involved in the early immune clearance process of the Omicron variant. Vitamin D insufficiency stimulates platelet activation and aggregation, which plays an important role not only during coagulation and thrombosis, but also during inflammation and immune responses (26). Our study found a positive correlation between platelets and serum 25(OH)D concentrations, and the sVD group had higher platelets within 8–14 days. This suggests that vitamin D sufficiency may reduce platelet activation and depletion. To date, there are still too many unknowns, and the specific roles and mechanisms of coagulation activation and platelets in the immune clearance of SARS-CoV-2 remain to be further explored.

Compared with other SARS-CoV-2 variants, the Omicron variant replicates more rapidly in the bronchi but less efficiently in the lung parenchyma (27, 28). Therefore, the Omicron variant is less likely to cause pneumonia, and the lesions are usually mild and difficult to measure accurately. In our investigation, the incidence of pneumonia in children infected with the Omicron subvariant BA.2 was basically consistent with this recent report (29). All were mild pneumonia lesions, mainly including GGOs and some small amount of consolidation. The ratio of total lesion volume to total lung volume on CT at admission was very small and did not differ significantly between the two groups. However, on repeat CT 1–2 weeks later, the sVD group showed more significant improvement in lesions characterized by a lower ratio of total lesion volume to total lung volume. Given the dynamic changes in laboratory data from the previous week, we speculate that vitamin D sufficiency may improve pneumonia lesions by accelerating viral clearance and balancing the inflammatory response.

There are several limitations in our longitudinal cohort study. First, due to the single-center, retrospective, non-randomized observational nature and small sample size, there are certain confounding factors. Second, the pediatric patients may be in different stages of COVID-19 upon admission. Third, some children's laboratory data have a small number of missing values at certain time periods, which may not reflect true differences. Fourth, this longitudinal study observes the dynamic changes of parameters over time periods rather than time points, which may have some influence on the final results. Therefore, these results should be carefully interpreted due to potential selection bias and residual confounding. Larger prospective randomized clinical trials may be required to provide further data support.

## Conclusions

Children with vitamin D insufficiency might have poorer clinical outcomes in Omicron subvariant BA.2 infection, especially in older pediatric patients. Vitamin D sufficiency, with a serum 25(OH)D concentration of at least 30 ng/mL, may accelerate viral clearance early in the course of COVID-19 and subsequently improve pneumonia lesions, and ultimately improve clinical outcomes. Further studies are needed to assess the effectiveness of supplements.

## Data availability statement

The original contributions presented in the study are included in the article/supplementary materials, further inquiries can be directed to the corresponding author.

## Ethics statement

This investigation involving human participants were reviewed and approved by the Ethics Committee of The Third People's Hospital of Shenzhen (Approval Number: 2022-012). Written informed consent for participation was not provided by the participants' legal guardians/next of kin because: Written informed consent from patients participating in this study was waived in accordance with the national legislation and the institutional requirements.

## Author contributions

DP: methodology, investigation, formal analysis, data curation, writing the original draft, and visualization. HH and ZL: investigation, formal analysis, and data curation. YL: conceptualization, investigation, review and editing, and supervision. ZL, YG, and HH: worked on conceptualization, formal analysis, investigation, and data curation. All authors have read and approved the final manuscript version to be submitted.

## Funding

This study was supported by the Shenzhen Longgang District Science and Technology Development Fund (LGKCXGZX2020002).

## Conflict of interest

The authors declare that the research was conducted in the absence of any commercial or financial relationships that could be construed as a potential conflict of interest.

## Publisher's note

All claims expressed in this article are solely those of the authors and do not necessarily represent those of their affiliated organizations, or those of the publisher, the editors and the reviewers. Any product that may be evaluated in this article, or claim that may be made by its manufacturer, is not guaranteed or endorsed by the publisher.
