# Supplementary figures and images for: Vitamin D levels and clinical outcomes of SARS-CoV-2 Omicron subvariant BA.2 in children: A longitudinal cohort study
Source: Front Nutr. 2022 Jul 25;9:960859. doi: 10.3389/fnut.2022.960859 (PMC9358048; doi:10.3389/fnut.2022.960859)

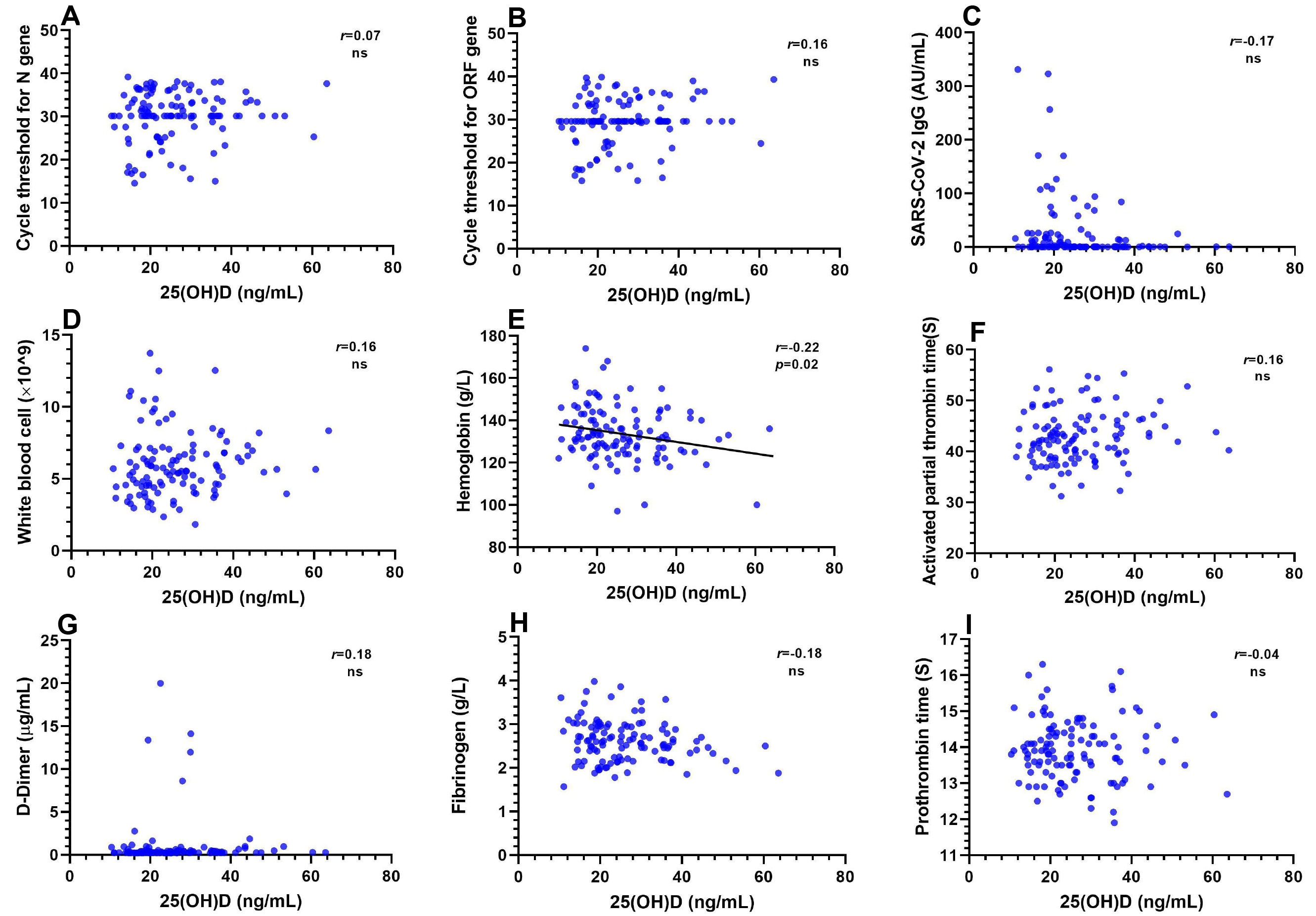

Supplement: Supplementary file 1 [file Image_1.JPEG]

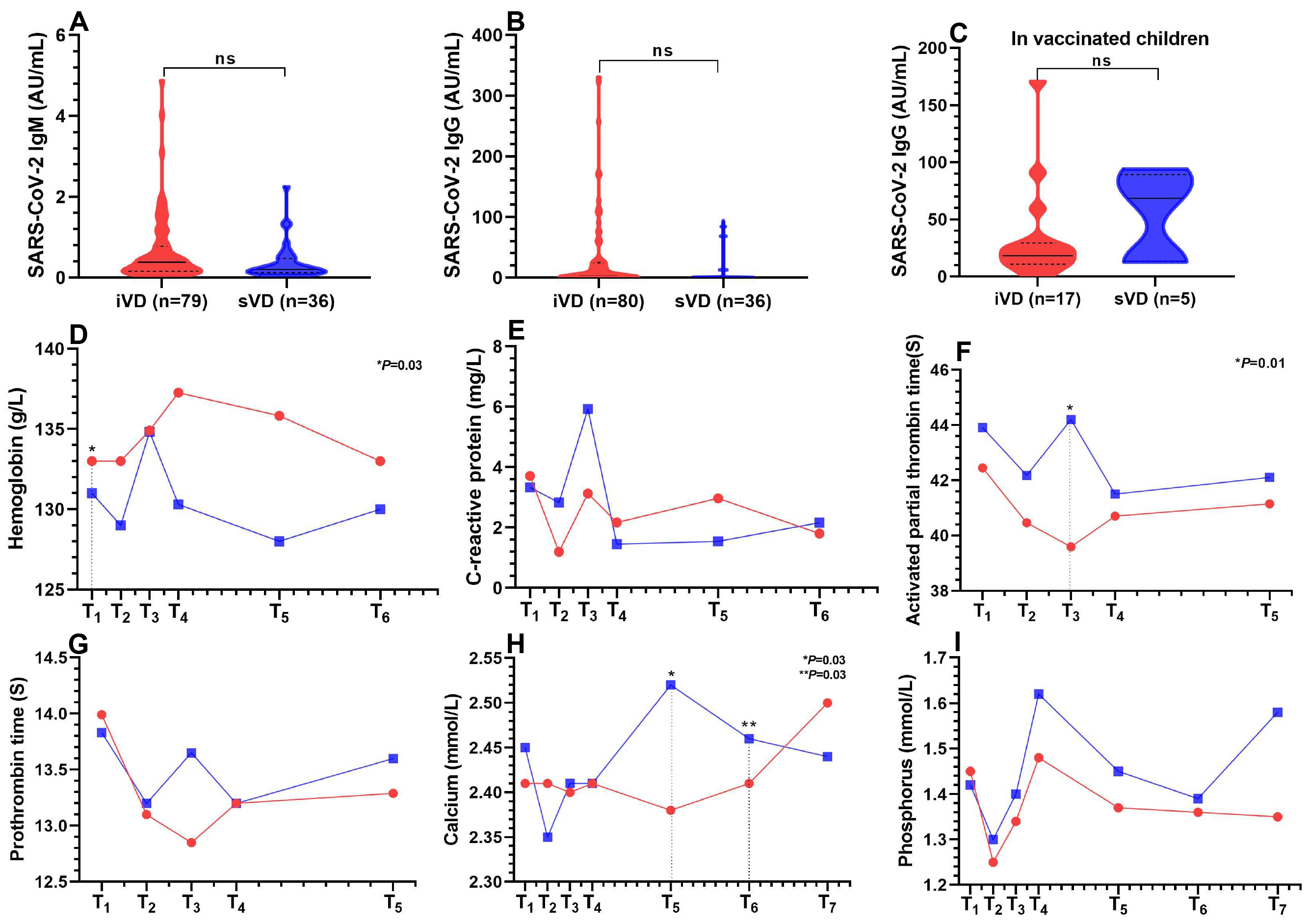

Supplement: Supplementary file 2 [file Image_2.JPEG]
