# Supplementary material for: Vitamin D levels and clinical outcomes of SARS-CoV-2 Omicron subvariant BA.2 in children: A longitudinal cohort study
Source: Front Nutr. 2022 Jul 25;9:960859. doi: 10.3389/fnut.2022.960859 (PMC9358048; doi:10.3389/fnut.2022.960859)
Supplement: Supplementary file 3 [file Table_1.pdf]

Table S1: Effects of vitamin D status on dynamic changes in laboratory parameters in children infected with SARS-CoV-2 subvariant BA.2

Time period (days)	Cycle threshold for N gene		*P* value
	The iVD group	The sVD group	
$T_1 \leq 1$ [median (IQR)]	18.5 (15.8-27.2) n=69	22.5 (15.2-28.9) n=30	0.75
$1 < T_2 \leq 3$	19.8 (18.5-22.7) n=15	30.6 (20.2-32.7) n=15	0.03
$3 < T_3 \leq 5$	24.5 (21.1-30.3) n=32	24.4 (20.5-32.6) n=13	0.69
$5 < T_4 \leq 7$ [mean±SD]	28.3±4.6 n=40	26.4±5.1 n=14	0.19
$7 < T_5 \leq 14$	33.1 (29.6-35.9) n=63	31.9 (27.8-34.2) n=30	0.15
$14 < T_6 \leq 21$	36.1 (33.9-37.3) n=49	35.9 (33.7-37.7) n=19	0.76
$21 < T_7 \leq 28$	36.5 (35.4-37.7) n=29	36.6 (34.3-37.8) n=12	0.79

Time period (days)	Cycle threshold for ORF1ab gene		*P* value
	The iVD group	The sVD group	
$T_1 \leq 1$ [median (IQR)]	20.1 (16.9-28.2) n=69	23.7 (16.8-28.7) n=31	0.56
$1 < T_2 \leq 3$	20.1 (19.4-23.3) n=15	31.4 (20.6-33.3) n=15	0.03
$3 < T_3 \leq 5$	24.2 (22.5-29.5) n=31	27 (20.1-33.4) n=13	0.61
$5 < T_4 \leq 7$ [mean±SD]	28.9±4.9 n=39	27.2±5.5 n=14	0.27
$7 < T_5 \leq 14$	33.4 (29.7-36.9) n=47	32.6 (28.1-37.7) n=30	0.75
$14 < T_6 \leq 21$	35.3 (33.8-37.9) n=23	37.9 (34.9-39.2) n=18	0.18
$21 < T_7 \leq 28$	37.9 (36.9-38.8) n=10	38.2 (35.8-38.4) n=7	0.38

Time period (days)	WBC (×10^9)		*P* value
	The iVD group	The sVD group	
$T_1 \leq 1$	5.52 (4.49-6.8) n=78	5.84 (4.63-7.14) n=35	0.34
$1 < T_2 \leq 3$	4.73 ± 1.25 n=15	6.31 ± 2.08 n=13	0.02
$3 < T_3 \leq 5$	5.39 (3.34-6.41) n=16	5.82 (4.86-7.12) n=5	0.45
$5 < T_4 \leq 7$	5.41 ± 0.82 n=11	7.46 ± 2.12 n=4	0.02
$7 < T_5 \leq 14$	6.84 ± 2.06 n=23	6.55 ± 1.33 n=4	0.79
$14 < T_6 \leq 21$	5.05 (4.64-6.18) n=11	7.15 (6.11-7.43) n=5	0.15

Time period (days)	Hemoglobin (g/L)		*P* value
	The iVD group	The sVD group	
$T_1 \leq 1$	133 (128-143.5) n=80	131 (123-138) n=36	0.03
$1 < T_2 \leq 3$	133 (127-138) n=17	129 (122.5-134.5) n=15	0.18
$3 < T_3 \leq 5$	134.9 ± 12.1 n=20	134.8 ± 14.4 n=6	0.99
$5 < T_4 \leq 7$	137.3 ± 5.5 n=11	130.3 ± 6.1 n=4	0.053
$7 < T_5 \leq 14$	135.8 ± 10.6 n=23	128 ± 11.6 n=4	0.19
$14 < T_6 \leq 21$	133 ± 7.3 n=11	130 ± 10.2 n=5	0.51

Time period (days)	Platelet (×10^9)		*P* value
	The iVD group	The sVD group	

$T_1 \leq 1$	217 (184-264) n=80	241 (195-300) n=36	0.16
$1 < T_2 \leq 3$	211 ± 58 n=17	220 ± 60 n=15	0.67
$3 < T_3 \leq 5$	201 (182-260) n=20	263 (252-271) n=6	0.11
$5 < T_4 \leq 7$	271 ± 61 n=11	269 ± 69 n=4	0.97
$7 < T_5 \leq 14$	301 ± 66 n=23	401 ± 32 n=4	0.007
$14 < T_6 \leq 21$	299 ± 56 n=11	375 ± 134 n=5	0.28

Time period (days)	Lymphocyte (×10^9)		*P* value
	The iVD group	The sVD group	
$T_1 \leq 1$	1.15 (0.74-1.85) n=78	1.61 (1.03-3.33) n=35	0.02
$1 < T_2 \leq 3$	1.95 (1.54-2.29) n=17	3.74 (2.34-5.04) n=15	0.001
$3 < T_3 \leq 5$	2.06 (1.9-3.22) n=20	3.93 (3.21-5.8) n=6	0.01
$5 < T_4 \leq 7$	2.73 (2.44-2.94) n=11	4.85 (4.02-6.93) n=4	0.02
$7 < T_5 \leq 14$	2.83 (2.49-3.63) n=23	4.39 (3.62-5.02) n=4	0.08
$14 < T_6 \leq 21$	2.5 (2.25-2.79) n=11	4.35 (3.76-4.73) n=5	0.07

Time period (days)	IL-6 (pg/mL)		*P* value
	The iVD group	The sVD group	
$T_1 \leq 1$	12.9 (7.5-21.2) n=78	18.4 (13.6-24.6) n=35	0.003
$1 < T_2 \leq 3$	9.6 (7.6-14.6) n=17	9.2 (8-17.7) n=12	0.81
$3 < T_3 \leq 5$	9.5 (7.6-17.1) n=17	16.2 (8.8-21.1) n=6	0.35
$5 < T_4 \leq 7$	10 (6.6-19) n=14	18.7 (11.3-22.4) n=4	0.33
$7 < T_5 \leq 14$	11.3 (7-20.5) n=16	8.2 (6.5-13) n=5	0.35
$14 < T_6 \leq 21$	8.8 ± 5.5 n=10	15.9 ± 10.7 n=4	0.12

Time period (days)	Procalcitonin (ng/mL)		*P* value
	The iVD group	The sVD group	
$T_1 \leq 1$	0.1 (0.06-0.19) n=78	0.15 (0.08-0.28) n=35	0.03
$1 < T_2 \leq 3$	0.07 ± 0.03 n=17	0.14 ± 0.09 n=12	0.03
$3 < T_3 \leq 5$	0.07 (0.05-0.12) n=17	0.21 (0.13-0.33) n=6	0.02
$5 < T_4 \leq 7$	0.06 (0.04-0.07) n=14	0.08 (0.05-0.08) n=4	0.38
$7 < T_5 \leq 14$	0.05 (0.04-0.06) n=16	0.05 (0.04-0.07) n=5	0.72
$14 < T_6 \leq 21$	0.04 ± 0.01 n=10	0.07 ± 0.04 n=4	0.18

Time period (days)	C-reactive protein (mg/L)		*P* value
	The iVD group	The sVD group	
$T_1 \leq 1$	3.7 (0.7-7.8) n=23	3.3 (1.1-5.1) n=6	0.48
$1 < T_2 \leq 3$	1.2 (0.5-5.5) n=13	2.8 (1.6-4.3) n=4	0.47
$3 < T_3 \leq 5$	3.1 (2.1-14.1) n=11	5.9 (2-14.8) n=5	0.91
$5 < T_4 \leq 7$	2.2 (0.8-3.5) n=12	1.5 (0.5-5.9) n=4	0.77
$7 < T_5 \leq 14$	3 (0.8-8.9) n=33	1.5 (0.6-3.1) n=13	0.17
$14 < T_6 \leq 21$	1.8 (0.6-5.7) n=53	2.2 (1.8-9.2) n=23	0.24
$21 < T_7 \leq 28$	3.8 (1.3-6.5) n=25	0.6 (0.5-6.8) n=3	0.73

Time period (days)	Activated partial thromboplastin time (S)		P value
	The iVD group	The sVD group	
$T_1 \leq 1$	42.4 ± 5.1 n=79	43.9 ± 5.4 n=35	0.16
$1 < T_2 \leq 3$	40.5 ± 2.7 n=13	42.2 ± 4.8 n=7	0.34
$3 < T_3 \leq 5$	40 ± 2.5 n=12	44.2 ± 2.5 n=4	0.01
$5 < T_4 \leq 7$	40.7 ± 4.1 n=10	41.5 ± 2 n=4	0.71
$7 < T_5 \leq 14$	41.1 ± 3.4 n=14	42.1 ± 6.4 n=4	0.68

Time period (days)	Prothrombin time (S)		P value
	The iVD group	The sVD group	
$T_1 \leq 1$	14 ± 0.8 n=79	13.8 ± 1.1 n=35	0.42
$1 < T_2 \leq 3$	13.1 ± 0.6 n=13	13.2 ± 0.5 n=7	0.96
$3 < T_3 \leq 5$	12.8 (12.6-13.3) n=12	13.7 (13.3-14) n=4	0.058
$5 < T_4 \leq 7$	13.2 ± 0.6 n=10	13.2 ± 0.6 n=4	0.94
$7 < T_5 \leq 14$	13.3 ± 0.7 n=14	13.6 ± 1.3 n=4	0.55

Time period (days)	Fibrinogen (g/L)		P value
	The iVD group	The sVD group	
$T_1 \leq 1$	2.6 ± 0.5 n=79	2.5 ± 0.4 n=35	0.33
$1 < T_2 \leq 3$	2.7 ± 0.3 n=13	2.1 ± 0.3 n=7	<0.001
$3 < T_3 \leq 5$	2.5 ± 0.4 n=12	2.3 ± 0.4 n=4	0.43
$5 < T_4 \leq 7$	2.6 ± 0.4 n=10	2.6 ± 0.2 n=4	0.85
$7 < T_5 \leq 14$	2.6 ± 0.5 n=14	2.7 ± 0.6 n=4	0.78

Time period (days)	D-dimer (μg/mL)		P value
	The iVD group	The sVD group	
$T_1 \leq 1$	0.27 (0.22-0.42) n=77	0.34 (0.26-0.49) n=35	0.03
$1 < T_2 \leq 3$	0.28 (0.22-0.43) n=13	0.5 (0.41-2.69) n=7	0.04
$3 < T_3 \leq 5$	0.23 (0.22-0.34) n=12	0.52 (0.23-0.81) n=4	0.23
$5 < T_4 \leq 7$	0.28 (0.22-0.33) n=10	0.55 (0.26-0.81) n=4	0.25
$7 < T_5 \leq 14$	0.22 (0.22-0.24) n=14	0.22 (0.22-0.23) n=4	0.36

Time period (days)	Calcium (mmol/L)		P value
	The iVD group	The sVD group	
$T_1 \leq 1$	2.41 (2.36-2.42) n=11	2.45 (2.41-2.52) n=6	0.15
$1 < T_2 \leq 3$	2.41 (2.38-2.54) n=12	2.35 (2.29-2.41) n=6	0.19
$3 < T_3 \leq 5$	2.4 (2.35-2.43) n=8	2.41 (2.41-2.43) n=5	0.29
$5 < T_4 \leq 7$	2.41 (2.36-2.43) n=10	2.41 (2.41-2.44) n=5	0.71
$7 < T_5 \leq 14$	2.38 (2.32-2.43) n=20	2.52 (2.41-2.54) n=5	0.03
$14 < T_6 \leq 21$	2.41 (2.33-2.45) n=26	2.46 (2.42-2.49) n=11	0.03
$21 < T_7 \leq 28$	2.5 (2.33-2.56) n=17	2.44 (2.35-2.54) n=5	0.58

Time period (days)	Phosphorus (mmol/L)		*P* value
	The iVD group	The sVD group	
$T_1 \leq 1$	1.45 ± 0.27 n=11	1.42 ± 0.31 n=6	0.69
$1 < T_2 \leq 3$	1.25 (1.15-1.4) n=12	1.3 (1.2-1.5) n=6	0.42
$3 < T_3 \leq 5$	1.34 (1.3-1.45) n=8	1.4 (1.1-1.4) n=5	0.88
$5 < T_4 \leq 7$	1.48 ± 0.24 n=10	1.62 ± 0.33 n=5	0.36
$7 < T_5 \leq 14$	1.37 (1.25-1.4) n=20	1.45 (1.39-1.6) n=5	0.73
$14 < T_6 \leq 21$	1.36 ± 0.24 n=26	1.39 ± 0.35 n=11	0.16
$21 < T_7 \leq 28$	1.35 ± 0.26 n=17	1.58 ± 0.42 n=5	0.14

Note: IL-6, interleukin-6; IQR, interquartile range; SARS-CoV-2, severe acute respiratory syndrome coronavirus 2; SD, standard deviation; WBC, white blood cell.
