# Supplementary material for: Vitamin D levels and clinical outcomes of SARS-CoV-2 Omicron subvariant BA.2 in children: A longitudinal cohort study
Source: Front Nutr. 2022 Jul 25;9:960859. doi: 10.3389/fnut.2022.960859 (PMC9358048; doi:10.3389/fnut.2022.960859)
Supplement: Supplementary file 4 [file Table_2.DOCX]

Table S2: Spearman correlation analysis of serum 25(OH)D concentrations with other clinical parameters in children infected with SARS-CoV-2 subvariant BA.2

| Y: [X=25(OH)D] | *r* | 95% CI | *P* value | Equation |
| --- | --- | --- | --- | --- |
| Age | -0.6 | -0.71 to -0.47 | <0.001 | Y = -0.276*X + 15.1 |
| SARS-CoV-2 IgM | -0.24 | -0.41 to -0.06 | 0.008 | Y = -0.03*X + 1.51 (Outlier included) |
|  | -0.22 | -0.4 to -0.04 | 0.02 | Y = -0.012*X + 0.88 (Outlier excluded) |
| Lymphocyte  Platelet  IL-6  Procalcitonin | 0.25 | 0.06 to 0.42 | 0.008 | Y = 0.035*X + 0.81 (Outlier included) |
|  | 0.23  0.19  0.3  0.29  0.27  0.25 | 0.05 to 0.4  0.005 to 0.37  0.11 to 0.46  0.11 to 0.46  0.08 to 0.43  0.07 to 0.42 | 0.01  0.04  0.001  0.002  0.004  0.006 | Y = 0.031*X + 0.86 (Outlier excluded)  Y = 0.76*X + 212  Y = 0.38*X + 7.85 (Outliers included)  Y = 0.25*X + 9.8 (Outliers excluded)  Y = 0.006*X + 0.11 (Outliers included)  Y = 0.003*X + 0.13 (Outliers excluded) |

Note: 25(OH)D, 25-hydroxyvitamin D; CI, confidence interval; IL-6, interleukin-6; SARS-CoV-2, severe acute respiratory syndrome coronavirus 2.
